# New EAF Slag Characterization Methodology for Strategic Metal Recovery

**DOI:** 10.3390/ma14061513

**Published:** 2021-03-19

**Authors:** Nour-Eddine Menad, Nassima Kana, Alain Seron, Ndue Kanari

**Affiliations:** 1 Waste and Raw Materials and Recycling Unit, Water, Environment Process and Analysis Department, BRGM, 3 Avenue Claude Guillemin, BP 36009, CEDEX, F-45060 Orléans, France; n.menad@brgm.fr (N.-E.M.); a.seron@brgm.fr (A.S.); 2 Institut des Matériaux Jean Rouxel, IMN, 2 Rue de la Houssinière, BP32229, CEDEX 3, F-44322 Nantes, France; kananassima@gmail.com; 3 GeoRessources, Université de Lorraine, CNRS, F-54000 Nancy, France

**Keywords:** EAF slag, characteristics, Mössbauer spectroscopy, valuable metals, recovery

## Abstract

The grown demand of current and future development of new technologies for high added value and strategic metals, such as molybdenum, vanadium, and chromium, and facing to the depletion of basic primary resources of these metals, the metal extraction and recovery from industrial by-products and wastes is a promising choice. Slag from the steelmaking sector contains a significant amount of metals; therefore, it must be considered to be an abundant secondary resource for several strategic materials, especially chromium. In this work, the generated slag from electric arc furnace (EAF) provided by the French steel industry was characterized by using multitude analytical techniques in order to determine the physico-chemical characteristics of the targeted slag. The revealed main crystallized phases are larnite (Ca_2_SiO_4_), magnetite (Fe_3_O_4_), srebrodolskite (Ca_2_Fe_2_O_5_), wüstite (FeO), maghemite (Fe_2.6_O_3_), hematite (Fe_2_O_3_), chromite [(Fe,Mg)Cr_2_O_4_], and quartz (SiO_2_). The collected slag sample contains about 34.1% iron (48.5% Fe_2_O_3_) and 3.5% chromium, whilst the vanadium contents is around 1500 ppm. The Mössbauer spectroscopy suggested that the non-magnetic fraction represents 42 wt% of the slag, while the remainder (58 wt%) is composed of magnetic components. The thermal treatment of steel slag up to 900 °C indicated that this solid is almost stable and few contained phases change their structures.

## 1. Introduction

Steel slag is an industrial by-product that is generated during steelmaking operations [1,2,3,4]. This material represents a significant potential economic resource due to its high content in strategic metals (SMs). The continuous increase in the manufacture of steel by melting scrap in the electric arc furnace (EAF) leads to the production of a large amount of EAF slag. Knowledge of the characteristics of slag is quite important in identifying the best process for recovering the strategic metals contained therein or its valorization as civil engineering materials. Yildirim and Prezzi [5] identify the chemical, mineralogical, and morphological properties of the various slag that is generated by the basic oxygen furnace (BOF) and EAF. According to them, the XRD patterns of BOF and EAF slags were very complex, with several overlapping peaks resulting from the many minerals that are present in the two slags. EAF slag has a chemical composition similar to that of BOF slag. The main chemical compounds of EAF slags can vary widely. As reported previously [5], oxide contents in EAF slags are typically FeO (10–40%), CaO (22–60%), SiO_2_ (6–34%), Al_2_O_3_ (3–14%), and MgO (3–13%).

According to Mostafaee [6], the metallurgical properties of the slag are strongly influenced by its microstructure at high temperature. The main constituent of the EAF slag is a melt composed of liquid oxides. Certain particles that present as spherical droplets were most likely projected from the molten bath into the slag layer during the melting process. It also contains solid particles of spinel with a diameter greater than 5 μm of general formula AB_2_O_4_, where A and B are divalent and trivalent cations, respectively. Such angular particles contain a high grade of chromium. The amount of spinel particles in the slag increases with increased chromium-oxide content in the slag. It is assumed that the small spinel particles were precipitated during the solidification of the slag sample. As reported by Abulikemu et al. [7], with the increase in the basicity of steel slags, the amount of precipitation of 2CaO·SiO_2_ increases and the area of the precipitated phase increases with a decreasing slag basicity.

The slag had superior low loss on ignition (0.01 wt%) due to the fact that it formed on top of molten steel at high temperature (1500 °C–1600 °C). The slag had close chemical composition with typical raw materials for ceramic tile production [8]. EAF slag can be used as blending material for Portland cement, by the hydration of Portland and EAF slag mixtures. The values of compressive strength of the mixtures containing 5 and 10 wt% EAF slag were near to those of the hardened neat Portland Cement Paste at most of the hydration ages, especially at the latest, according to Hekal et al. [9].

Petrakis et al. [10] evaluated the particle size distribution (PSD) of crushed Ferro-nickel slag at different residence time. Then, selected products were alkali activated in order to investigate the effect of particle size on the compressive strength of the produced alkali activated materials (AAMs). The results show that the grinding of slag exhibits non-first-order behavior and the reduction rate of each size is time dependent. The authors concluded that the AAMs that are produced with fine particles of raw slag acquire higher compressive strength reaching the maximum value of 60.8 MPa. The physical and mechanical properties of EAF slag are very important to study because of their utilization as a substitute for natural mineral aggregates in the production of asphalt mixtures in road construction [11].

Selected studies [12,13,14,15,16,17,18,19,20,21] of numerous recent research works reported to the scientific journal *Materials* were devoted to the use and incorporation of steel slags in road construction materials and concrete. The BOF slag incorporated in the asphalt mixture is advantageous for improving the ability of this mixed material to resist the deformation and enhancing the stability of structure, according to Ye et al. [12]. Skaf et al. [13] produced a porous asphalt mixture with 100% EAF slag aggregates that met the current standards for sustainable and environmentally friendly mixtures. Dondi et al. [15] stated that the asphalt mixture containing 30 % of steel slag is characterized by high stability and stiffness. Liu et al. [14] suggested that pits and grooves on the surface of steel-slag particles provided a skeleton-like function for the bitumen–steel slag aggregate interface improving the adhesion strength of the bitumen–steel slag aggregate interface.

Parron-Rubio et al. [16] reported replacing cement by 25% slag, which is considered to be a good strategy for reducing cement consumption and solving the waste problem. The use of steel slag as a partial replacement for cement could make a significant contribution to the steel industry and help to reduce CO_2_ emissions [17]. The partial replacement of cement by various steel and blast furnace slags was also recently reported [18,19,20]. Li et al. [21] studied the effects of steel slag and expansive agents on the properties of ultra-high performance concrete, and they stated that 15% slag and 5% expansive agents are required proportions to produce a good quality of targeted concrete.

However, a few studies [1,4,22,23,24,25] were focused on the metals’ separation and recovery from steel slags, and several findings are summarized early [4]. In this context, the main objective of this work is to know EAF slag characteristics in order to facilitate recovering of the steel slag bearing SMs using an appropriate technology and their utilization for both eco-compatible and cost-effective uses. To do that, a protocol of physico-chemical characterization of steel slag that is generated from EAF process is developed. The study carried out is part of the French research project (hydrometallurgy and phyto-management approaches for steel slags management) funded by the French National Research Agency. It concerns the recovery of valuable metals from steel slag using innovative technologies.

## 2. Material and Methods

**Figure 1:**
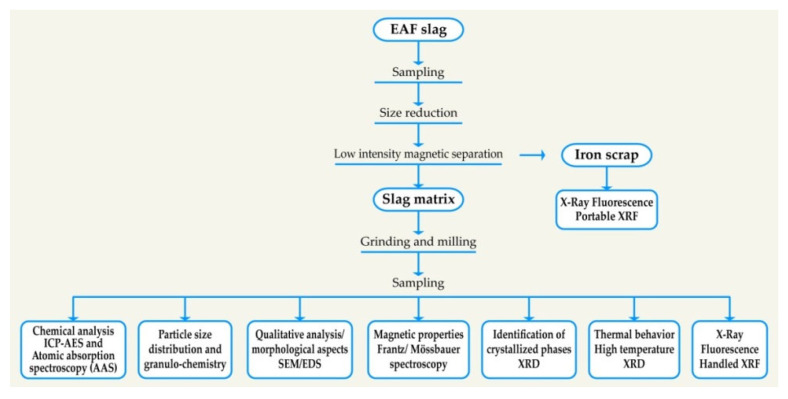
Experimental protocol for characterization of electric arc furnace (EAF) slag.

A French steel company supplied the EAF slag (Châteauneuf, France) that was studied in this work. A sampling protocol was employed to collect a representative sample. Figure 1 illustrates the experimental protocol used to characterize this slag. Different techniques have been performed to identify in detail the characteristics of this slag. Morphological aspects and semi-quantitative analyzes were carried out using scanning electron microscopy through energy-dispersive spectroscopy (SEM/EDS) (JEOL Ltd, Tokyo, Japan). The chemical composition was determined using inductively coupled plasma atomic emission spectroscopy (ICP-AES) (Ultima 2, Horiba Tokyo, Japan) and X-ray fluorescence (XRF) (Olympus IMS, Waltham, MA, USA). Physico-chemical properties, such as magnetic properties, have been also determined. The particle size distribution and the granulochemistry were taken into account in this work. The electro-fragmentation technique was carried out in order to study the grindability of the slag studied. The magnetic properties were determined using Mössbauer spectroscopy and a Frantz magnetic separator. The thermal behavior of the slag was studied by high temperature X-ray diffraction (HT-XRD) (Bruker, Karlsruhe, Germany) to determine the stability of the crystalline phases of the slag in the temperature range from 30 °C to 900 °C under air and nitrogen atmosphere.

The equipment and apparatus used for various analysis as well as the analytic protocols were thoroughly described earlier [26,27].

## 3. Results and Discussion

### 3.1. Chemical Composition of EAF Slag

The steel slag sample was quartered and then analyzed by handheld XRF and ICP-AES. Table 1 summarizes the results of the overall chemical analysis. It is important to emphasize that, depending on the fractions considered, the slag sample collected may contain about 3.4% of total Cr and approximately 34.1% Fe (48.5% of Fe_2_O_3_). The vanadium content is close to 1500 ppm (portable XRF results). Furthermore, the ICP-AES results confirm that the chromium content varies from 3 to 4%, and that of vanadium is between 1000 and 1400 ppm. Calcium is present at 21% (29.5% CaO).

### 3.2. Particle Size Distribution

**Figure 2:**
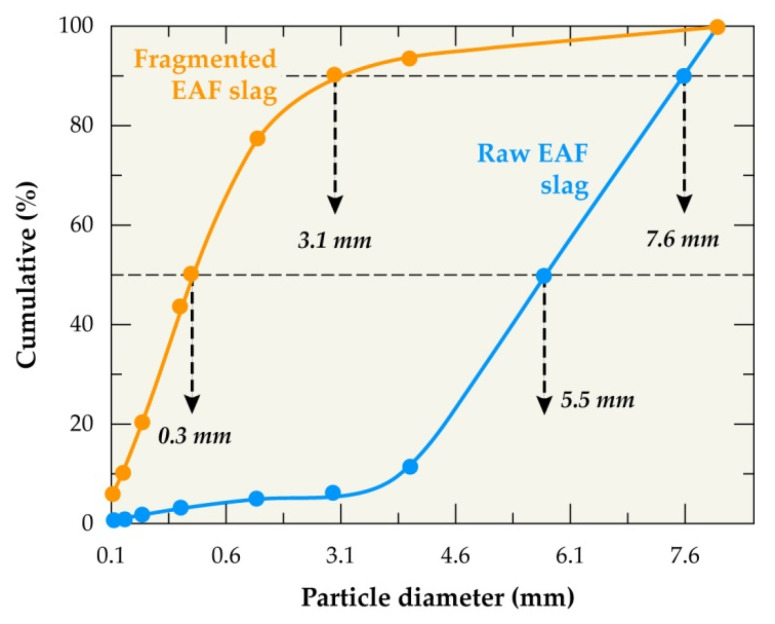
Particle size distribution of raw EAF slag and electro-fragmented EAF slag.

The particle size distribution was carried out on both representative samples of EAF raw slag and fragmented slag by an electro-fragmentation technique. The following sieves named: 8 mm, 4 mm, 2 mm, 1 mm, 0.5 mm, 0.25 mm, 0.125 mm, 0.063 mm, and 0.03 mm were used for the sieving process. Figure 2 shows the results. It can be seen that the d_50_ of raw slag was determined at 5.5 mm and, for the fragmented slag, was about 0.3 mm. In addition, the d_90_ of raw EAF slag is about 7.6 mm and that of the fragmented slag is approximately 3.1 mm.

### 3.3. Granulo-Chemistry of Raw and Fragmented Slag

**Figure 3:**
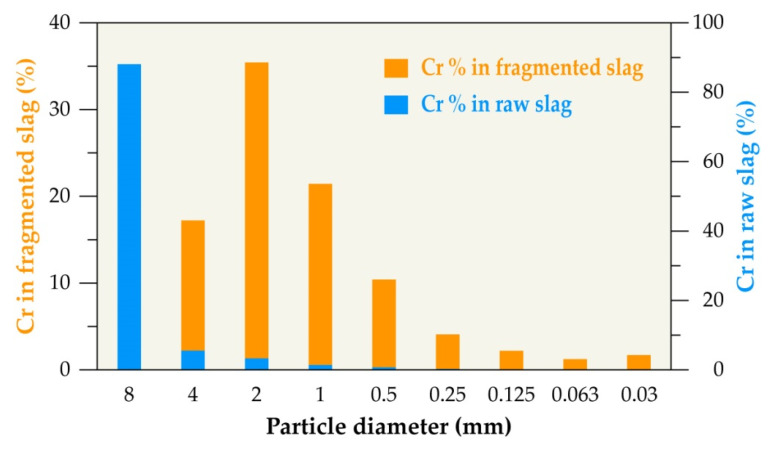
Chromium distribution in different particle size fractions of raw and fragmented slag.

**Figure 4:**
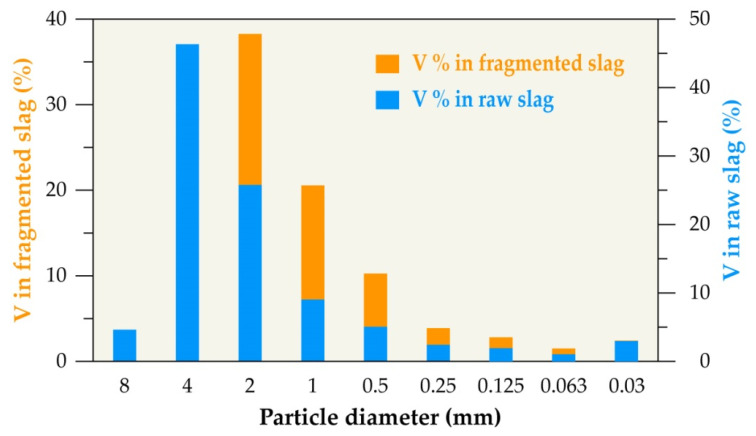
Vanadium distribution in different particle size fractions of raw and fragmented slag.

The particle size fractions that were obtained from the sieving process of both raw and electro-fragmented slags were analyzed by ICP-AES. The distribution of chromium and vanadium contained in the studied slag was calculated and the results are presented in Figure 3 and Figure 4, respectively.

Figure 3 shows that the chromium is accumulated in the coarse fraction (8 mm) of the raw slag; this is because of non-liberated chromium bearing phases and the average chromium content, as shown by chemical analysis, is in the range of 3% to 4%. However, in the fragmented slag, it is distributed in different particle fractions and mainly accumulated in the 2 mm particle size fraction. It represents approximately 35%.

The vanadium content is further accumulated in a coarse size fraction of raw slag (−8/+4 mm) and distributed into three main size fractions of −4/+2, −2/+1, and −1/+0.5 mm representing 45%, 25%, and 15%, respectively, of the fragmented slag, as shown in Figure 4.

### 3.4. Mineralogical Composition of EAF Slag

**Figure 5:**
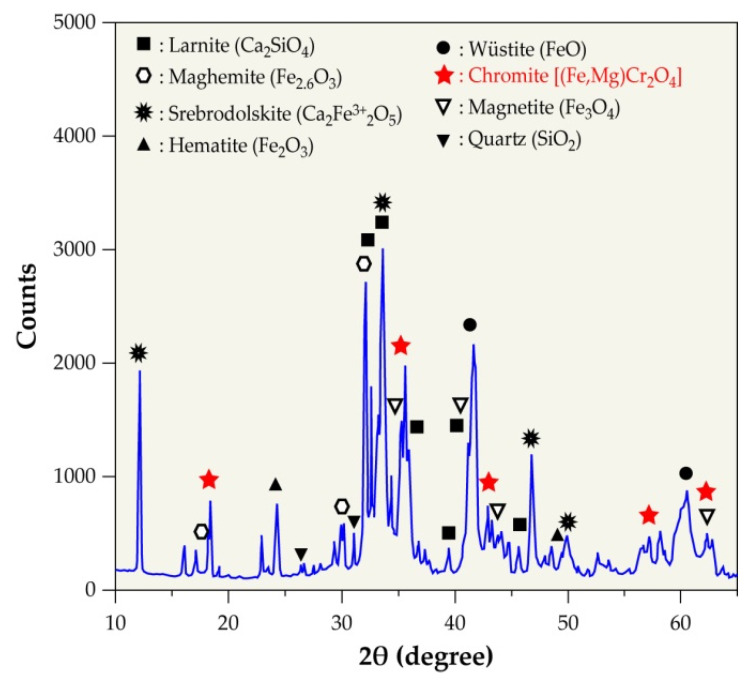
X-ray diffraction (XRD) pattern of EAF slags.

X-ray diffraction (XRD) determined the mineralogical composition of the slag sample, and Figure 5 shows the obtained result. The main crystalline phases identified are larnite (Ca_2_SiO_4_), srebrodolskite (Ca_2_Fe_2_O_5_), hematite (Fe_2_O_3_), magnetite (Fe_3_O_4_), maghemite (Fe_2.6_O_3_), wüstite (FeO), iron chromite (FeCr_2_O_4_), magnesiochromite (MgCr_2_O_4_), and quartz (SiO_2_). Note that the chromium bearing phases belong to the spinel family and they have a same structure and similar lattice parameters. The partial substitution of Fe^2+^ by Mg^2+^ often occurred [28,29]; hence, the chromium bearing phases (i.e., FeCr_2_O_4_ and MgCr_2_O_4_) in the diffractogram are noted as chromite [(Fe,Mg)Cr_2_O_4_].

### 3.5. Morphological Aspects and Quality of EAF Slag

**Figure 6:**
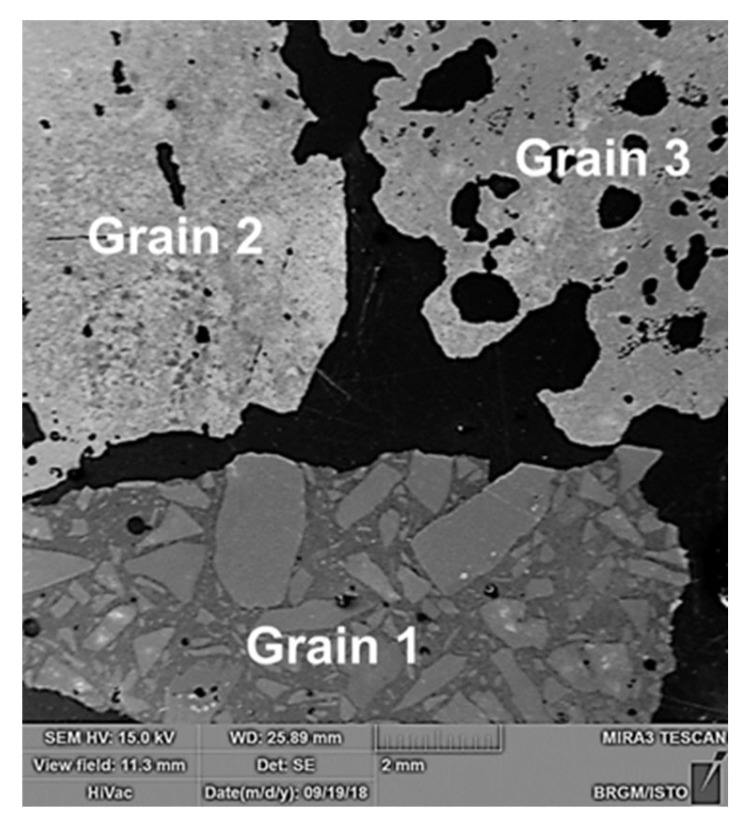

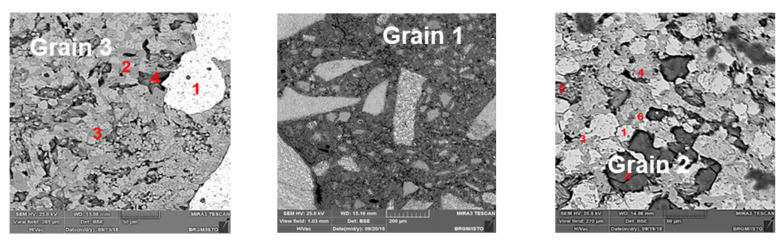
Morphological characterization by scanning electron microscopy of three different grains of the EAF slag sample. Numbers 1 to 6 indicate the spots for EDS analysis [26].

The investigated slag sample was characterized from a morphological and chemical point of view by scanning electron microscopy (SEM). Several grains in this sample were studied in order to take its heterogeneity into account, which is clearly apparent (grain 1, grain 2, and grain 3 of Figure 6). Certain grains consist of an agglomerate of particles whose size is between 0.3 and 2 mm, embedded in a fine matrix, while other grains are formed of a matrix fine dotted with elements of porosity, as shown in Figure 6.

**Figure 7:**
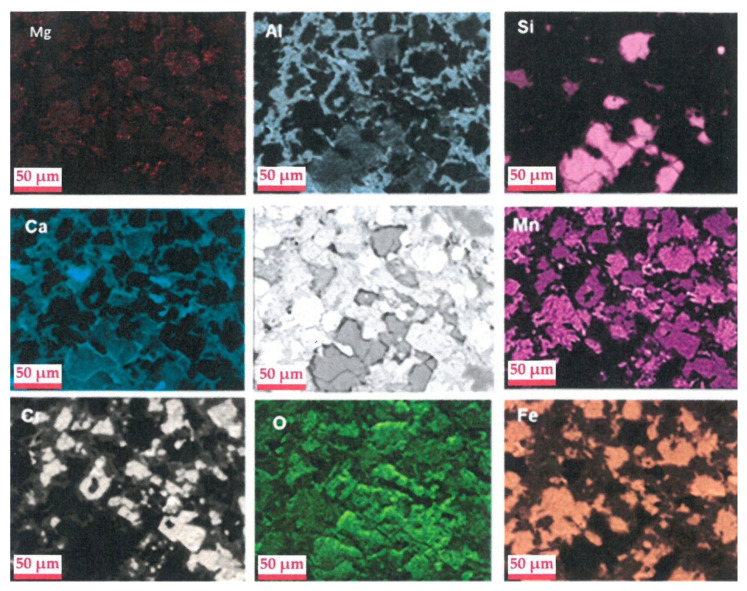
Energy dispersive spectroscopy (EDS) mapping from scanning electron microscopy of the elements Al, Si, Ca, Cr, Mn, Fe, and O in EAF slag.

The study by EDS mapping of spatial distribution of the elements Al, Si, Ca, Cr, Mn, Fe, and O (Figure 7) clearly establishes some correlations between some elements, which provide information on the minerals that comprise the slag. Moreover, such studies may also provide information on the nature of the minerals containing chromium. Indeed, the EDS mapping study clearly establishes a partial correlation between Cr and Mn concerning certain grains moderately charged with Mn, but highly charged with Cr. In addition, grains weakly loaded with Cr and highly loaded with Ca also exist. Moreover, in view of a correlation between Al, Ca, and Cr in grains weakly charged with chromium, it is probable that an unknown phase of alumino-calcium type is also one of the chromium bearing minerals that is not revealed by XRD analysis. Chromium is also often associated to iron, most likely as FeCr_2_O_4_ (chromite), as revealed by XRD.

A correlation between calcium and silica exists that most likely indicates the presence of a larnite like phase (Ca_2_SiO_4_), which does not appear in view of the distribution of the elements carrying chromium.

In addition, manganese is also bound to iron in the particles that are richest in Fe and Mn. Likewise, the calcium and aluminum concentrations are correlated in particles sometimes containing iron.

The spatial distribution of all these elements is also logically correlated with that of oxygen, with the mineral phases mentioned being oxides compounds.

**Figure 8:**
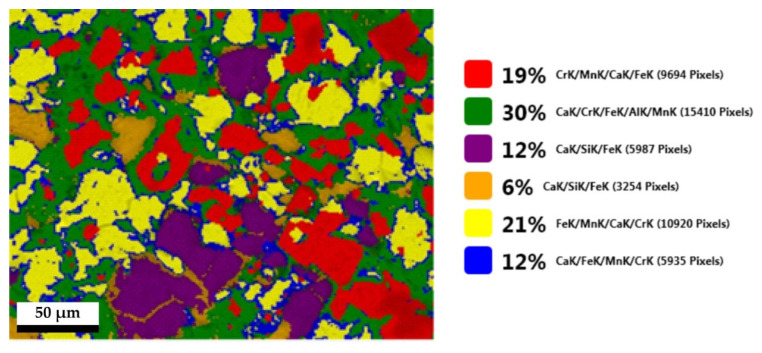
Distribution of phases, recalculated from EDS mapping from scanning electron microscopy of the elements Al, Si, Ca, Cr, Mn, Fe, and O in EAF slag.

The distribution of recalculated phases (Figure 8), from the mapping data, confirms these observations.

### 3.6. Magnetic Properties of EAF Slag

#### 3.6.1. Magnetic Separation (Frantz)

The Frantz separation technique makes it possible to separate the magnetic and non-magnetic phases according to the intensity of the applied magnetic field. This technique is implemented while using an isodynamic magnetic separator of the Frantz type. The principle of the measurement consists in moving the slag (d = 50–125 µm) in the magnetic field on a vibrating strip. The strip is divided approximately halfway into two terminal channels in order to recover the two magnetic and non-magnetic fractions. The magnetic grains attracted by the field enter the outermost channel relative to the body of the device, the non-magnetic grains pass through the other channel, and the two fractions resulting from the separation are collected at the end of the strip in two recovery bins.

In addition to the intensity of the current, various variables make it possible to modify the separation conditions, such as the lateral inclination and the longitudinal slope. The different intensities imposed in the equipment: 0.5A, 0.1A, and 0.2A, make it possible to modulate the magnetic field: 90, 110, 160 G, respectively. These magnetic fields are measured using a portable gauss-meter (AC/DC Magnetic Meter PCE-MFM 3000, PCE Americas Inc., Jupiter / Palm Beach, FL, USA). For our work, the experiments were carried out with a lateral inclination and a longitudinal slope of 10° and 40°, respectively. Subsequently, XRD was used to analyze the six collected fractions (magnetic and non-magnetic).

The investigated samples of steel slag were separated in nonmagnetic and magnetic fractions by Frantz magnetic separator at the following intensities: 0.05A, 0.1A, and 0.2A. The goal of this essay is to characterize the magnetic properties of the steel slag.

The magnetic and nonmagnetic fractions of slag that were obtained from magnetic separator Frantz have been examined by the XRD technique. It was observed that wustite (FeO) appears to be more concentrated in the nonmagnetic fraction. We conclude that the separation is successful, knowing that the wüstite is a non-magnetic phase. Moreover, during separation at 0.1A and 0.2A; the proportion of larnite (Ca_2_SiO_4_) significantly increases in the nonmagnetic fractions. It is important to note that the magnetic separation can be very advantageous in separating the magnetic and non-magnetic phases from the slag and, therefore, for concentrating the phase bearing metals of interest.

#### 3.6.2. Mössbauer Spectroscopy

The use of Mössbauer spectrometry makes it possible to determine the speciation and the valence of iron in the slag. Iron is mainly found in two groups of minerals: silicate (or clay) materials and oxides, as previously shown by XRD. The signature and discrimination of iron in these two groups of minerals can be relatively easy based on a study as a function of temperature [30].

Indeed, over a wide temperature range, from ambient down to around 10 K, silicate materials, in particular clays, are in the form of paramagnetic doublets. It can contain iron in the divalent and trivalent state, whereas most of the iron oxides are magnetically ordered when the crystallographic order is respected (with spectra in the form of sextuplets) with iron in the trivalent state [31]. However, crystal disorder problems can disturb the Mössbauer signature of oxides in natural samples, due to the phenomenon of superparamagnetic that is linked to particle size and therefore to the presence of nanophases. This constraint will clearly influence the characterization by XRD of nanoscopic phases, making it very difficult for comparison/correlation between the XRD results and those obtained by Mössbauer spectrometry.

**Figure 9:**
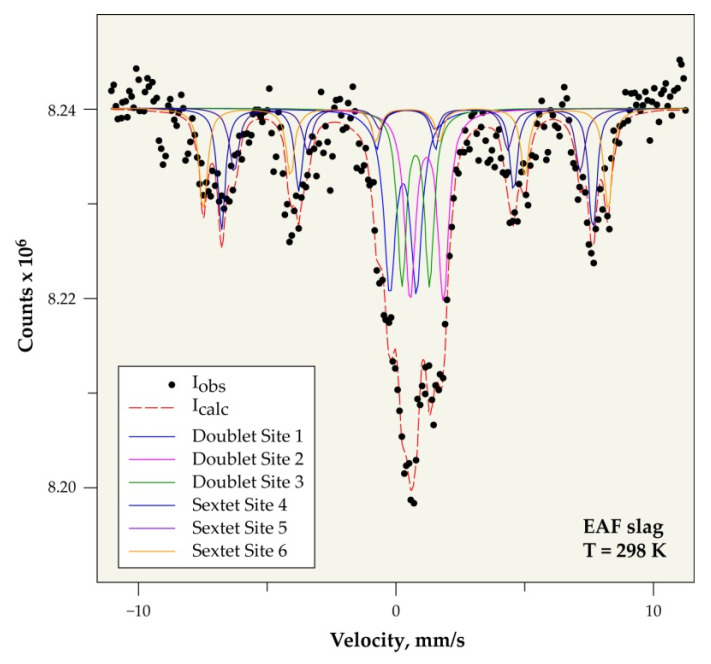
Mössbauer spectrum of slag at room temperature.

**Figure 10:**
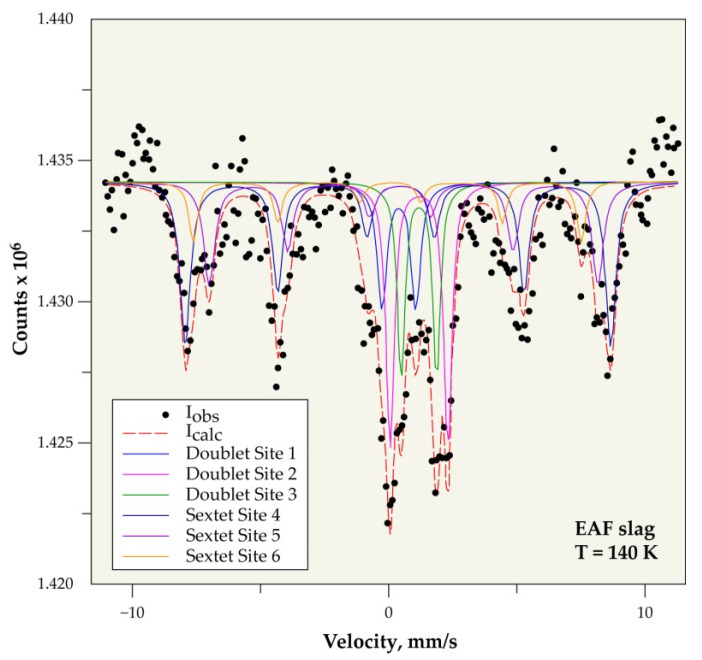
Mössbauer spectrum of slag at 140 K.

This difficulty can be overcome by recording the Mössbauer spectra at a very low temperature. This is probably what happens during the recording for the slag sample between the results that were obtained at room temperature (Figure 9) and those obtained at 140 K (Figure 10), where the overall paramagnetic contribution goes from 54% to 42% when the temperature drops.

When considering that the most reliable results are the results given at low temperature, the non-magnetic fraction seems to represent 42 wt% of the slag, while the magnetic fraction represents 58 wt%.

Table 2 and Table 3 give, respectively, at room temperature and at 140 K, the center shift (CS) values relative to that of the standard α-Fe at room temperature and the quadrupole splitting (Δ) characterizing the valence of the iron site. The hyperfine magnetic field (H) and the relative abundance (AR) compared to the global iron content in the slag.

### 3.7. Thermal Behavior of EAF Slag

Another possibility for treating steel slag is to modify its chemistry before its physical treatment. For such a treatment, the ground EAF slag was characterized by high temperature XRD (HT-XRD) under air and nitrogen atmospheres (1 bar) in a temperature range from 30 °C to 900 °C. The aim of this study is to identify the transformation of certain contained phases and, more particularly, the iron-rich phases to facilitate (by modifying their magnetic properties) their extractions by physical sorting.

**Figure 11:**
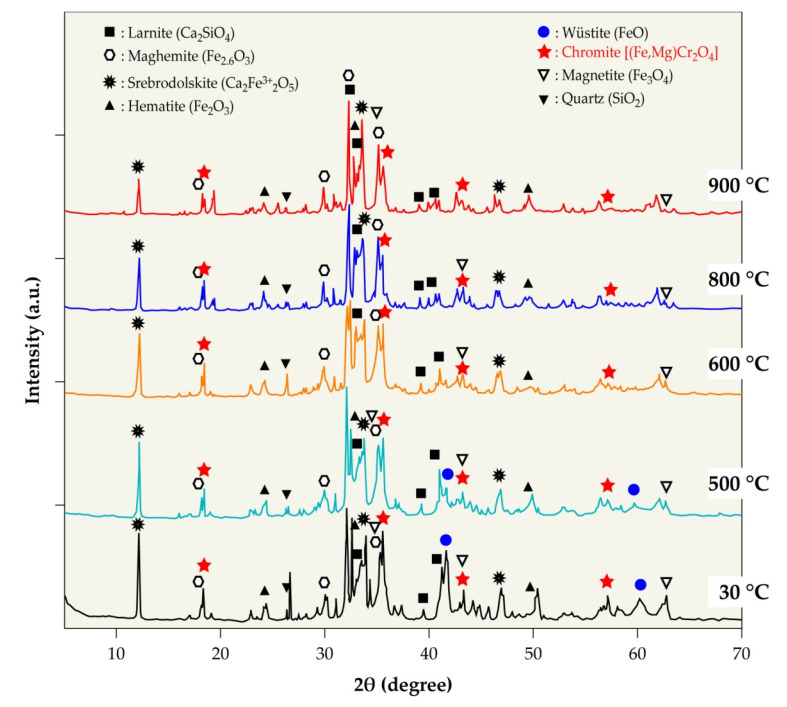
High temperature X-ray diffraction (HT-XRD) patterns of slag heated from 30 to 900 °C in an air atmosphere (1 bar).

Figure 11 and Table 4 present the results of the HT-XRD of products that are issued from EAF slag treated in air. The wüstite (FeO) crystalline phase, identified in the raw slag, disappeared around 600 °C, probably due to its oxidation into higher oxides and/or to its transformation into silicate. The beta modification crystalline of quartz is observed starting at 500 °C. Changes in the mesh parameters of certain phases (larnite and srebrodolskite) appeared at temperatures higher than 600 °C. It can be noted that a calcium silicate phase (CaSiO_3_) most likely wollastonite crystallizes at temperatures higher than or equal to 800 °C due to the reaction of calcium oxide with silica [32].

The XRD results obtained for the treatment of the EAF slag under nitrogen showed trends that were quite similar to those obtained during the treatment in air atmosphere.

### 3.8. Thermochemical Route for the Strategic Metal Recovery from EAF Slag

The results obtained from various above-mentioned analyses applied to the steel slag indicated that it is a complex solid. Moreover, that allows for pointing out that the targeted metals (especially chromium) have been disseminated in various phases [e.g., (Fe,Mg)Cr_2_O_4_] belonging to the spinel group considered to be non-reactive with respect to several chemical agents. However, the thermodynamic calculation and the literature review [26] suggest that alkali metal hydroxides (NaOH and KOH) and mixed with alkali metal carbonates (NaOH–Na_2_CO_3_ and KOH–K_2_CO_3_) are suitable chemicals for reacting with the targeted metal compounds at moderate temperatures leading to the synthesis of chromate (CrO_4_^2−^), vanadate (VO_4_^3−^), and molybdate (MoO_4_^2−^) of the alkali metals, which are known for the significant water solubility character.

When considering such a calculation, after the elimination of the ferromagnetic fraction using low magnetic intensity, the slag was co-ground with the selected alkali agents at reactant to a slag ratio equal to 1. The objective of the co-grinding was to assure an intimate mixture and a high surface contact between slag components and the chosen reagent. According to the thermodynamic predictions for (Cr, V, Mo)–(Na, K)–O systems and preliminary experimental tests [26], the co-grounded materials were subjected to roasting at three fixed temperatures, named 400, 600, and 800 °C. The obtained solid products were leached by water to recover the synthetized metal salts by leaching. Besides the temperature influence, the effect of co-grinding duration and impact of the carbonate on the recovery extent of Cr, V, and Mo was evaluated. Table 5 summarizes the best conditions for the recovery yield of these three metals.

The recovery yields of chromium and molybdenum seem to be at a satisfactory level by using co-grinding, of steel slag and alkali reagent, for 1 h and roasting the obtained mixture at 800 °C. Regarding vanadium, the best recovery yield was observed for a roasting temperature close to 600 °C, and it was no more than 62%. As expected, the selective separation and extraction of these metals from leachate will be the next step of this study.

## 4. Conclusions

These co-products have difficult times regarding their storage and use conditions due to the lack of management of the steel slags allowing for their recycling. In recent years, several applications have been identified to allow such valorization. One of them could be to use slags as a secondary resource because they contain a high content of strategic metals, in particular Cr. A methodology for the characterization of such byproducts generating from steel works has been developed in order to facilitate the development of the process to recover metals from slags. Such mechanical treatment has been completed with a strategy of preliminary modifications of the slag involving the separation of magnetic and non-magnetic fractions, but also modifications of mineral phases using heat-treatment under air or nitrogen atmosphere.

The slag that was generated from electric arc furnace provided by the French steel industry was characterized by different conventional techniques. The chemical analysis performed by ICP-AES and portable XRF shows that the investigated sample of slag contains 3.5% chromium and approximately 48.5% of Fe_2_O_3_.

The vanadium content is close to 1500 ppm (XRF results). Furthermore, the ICP-AES results confirm that the chromium content varies from 3 to 4% and that of vanadium is between 1000 and 1400 ppm, which is in a fairly good agreement with the analyzes carried out using XRF equipment.

XRD analysis allowed for the identification of the following main crystalline phases: larnite (Ca_2_SiO_4_), srebrodolskite (Ca_2_Fe_2_O_5_), hematite (Fe_2_O_3_), magnetite (Fe_3_O_4_), maghemite (Fe_2.6_O_3_), and wüstite (FeO). Compounds carrying chromium [(Fe,Mg)Cr_2_O_4_] have also been detected, although this element is also dispersed in various other mineral phases.

The non-magnetic fraction represents 42 wt% of the investigated slag sample, while the remainder (58 wt%) is composed of magnetic phases, according to the results that were obtained from Mössbauer spectroscopy.

The magnetic separation is very advantageous in operating the elimination of the ferromagnetic and non-magnetic phases from the slag and, therefore, for concentrating the phases bearing metals of interest, especially chromium.

The thermal treatment up to 900 °C shows that the studied EAF slag is almost stable, and a few phases may transform in other structures, including iron, calcium, and silicon oxides.

The proposed thermochemical process involves co-grinding the selected fraction of the EAF slag with alkali metal reagents followed by roasting at temperatures approaching 800 °C. Over 96 % of chromium and molybdenum are transformed, respectively, into chromate (CrO_4_^2−^) and molybdate (MoO_4_^2−^) of alkali metals that are separated from the roasted EAF slag by leaching with water.

## Figures and Tables

**Table 1:** Chemical composition of EAF slag, obtained by inductively coupled plasma atomic emission spectroscopy (ICP-AES).

Unit	Fe_2_O_3_	Al_2_O_3_	CaO	Cr	MgO	MnO	SiO_2_
%	48.5	5.2	29.5	3.4	4.3	5.2	5.7
Unit	Pb	Zn	Ni	Cu	Mo	V	P_2_O_5_
mg/kg	23	677	743	10	154	1450	2556

**Table 2:** Mössbauer parameters and distribution of magnetic and paramagnetic fractions from Mössbauer spectroscopy results of EAF slag at room temperature (298 K).

Iron Site	CS (mm/s)	Δ (mm/s)	H (T)	AR (%)
Site 1: paramagnetic ferric	0.27	1.03		20
Site 2: paramagnetic ferrous	1.19	1.3		19
Site 3: paramagnetic ferrous	0.76	1.07		15
Magnetic sextet site 1	0.41	0.02	44.7	18
Magnetic sextet site 2	0.44	−0.01	41.7	12
Magnetic sextet site 3	0.41	−0.038	48.7	16

**Table 3:** Mössbauer parameters and distribution of magnetic and paramagnetic fractions from Mössbauer spectroscopy results of EAF slag at 140 K.

Iron Site	CS (mm/s)	Δ (mm/s)	H (T)	AR (%)
Site 1: paramagnetic ferric	0.39	1.31		10
Site 2: paramagnetic ferrous	1.18	2.25		19
Site 3: paramagnetic ferrous	1.17	1.38		13
Magnetic sextet site 1 (Maghemite)	0.41	−0.05	51.5	29
Magnetic sextet site 2	0.51	0.06	47	20
Magnetic sextet site 3	0.55	−0.05	46.9	9

**Table 4:** XRD evaluation of EAF slag modifications during treatment in air from 30 to 900 °C.

T (°C)	Maghemite/Chromite	Larnite	Magnetite	Srebrodolskite	Hematite	Wustite	Quartz	CaSiO_3_
30	+++ ^1^	+++	+++	+++	+++	+++	+++	--- ^2^
500	+++	+++	+++	+++	+++	+++	+++	---
600	+++	+++	+++	+++	+++	---	+++	---
800	+++	probable modification of the mesh parameters	+++	structural modification: transition to a brownmillerite type structure	+++	---	+++	+++
900	+++	probable modification of the mesh parameters	+++	structural modification: transition to a brownmillerite type structure	+++	---	+++	+++

^1^ Identified phases; ^2^ Non-identified phases.

**Table 5:** Summary of the best conditions for the recovery of Cr, V, and Mo from EAF slag.

Metal	Co-Grinding Time, (Min)	Reagent	Roasting Temperature (°C)	Recovery Yield (%)
Chromium	60	NaOH–Na_2_CO_3_	800	97.5
Vanadium	60	KOH–Na_2_CO_3_	600	62.5
Molybdenum	60	KOH	800	96.3

## Data Availability

The data presented in this study are available on request from the corresponding author.
